# Multilevel Intervention Stepped Wedge Designs (MLI-SWDs)

**DOI:** 10.1007/s11121-024-01657-y

**Published:** 2024-05-15

**Authors:** John Sperger, Michael R. Kosorok, Laura Linnan, Shawn M. Kneipp

**Affiliations:** 1 Department of Biostatistics, Gillings School of Global Public Health, The University of North Carolina at Chapel Hill, Chapel Hill, USA; 2 Department of Health Behavior, Gillings School of Global Public Health, The University of North Carolina at Chapel Hill, Chapel Hill, USA; 3 School of Nursing, The University of North Carolina at Chapel Hill, Chapel Hill, USA

**Keywords:** Experimental design, Multilevel interventions, Stepped wedge, Cluster randomized trials, Unemployment

## Abstract

Multilevel interventions (MLIs) hold promise for reducing health inequities by intervening at multiple types of social determinants of health consistent with the socioecological model of health. In spite of their potential, methodological challenges related to study design compounded by a lack of tools for sample size calculation inhibit their development. We help address this gap by proposing the Multilevel Intervention Stepped Wedge Design (MLI-SWD), a hybrid experimental design which combines cluster-level (CL) randomization using a Stepped Wedge design (SWD) with independent individual-level (IL) randomization. The MLI-SWD is suitable for MLIs where the IL intervention has a low risk of interference between individuals in the same cluster, and it enables estimation of the component IL and CL treatment effects, their interaction, and the combined intervention effect. The MLI-SWD accommodates cross-sectional and cohort designs as well as both incomplete (clusters are not observed in every study period) and complete observation patterns. We adapt recent work using generalized estimating equations for SWD sample size calculation to the multilevel setting and provide an R package for power and sample size calculation. Furthermore, motivated by our experiences with the ongoing *NC Works 4 Health* study, we consider how to apply the MLI-SWD when individuals join clusters over the course of the study. This situation arises when unemployment MLIs include IL interventions that are delivered while the individual is unemployed. This extension requires carefully considering whether the study interventions will satisfy additional causal assumptions but could permit randomization in new settings.

The National Institute of Minority Health and Health Disparities (NIMHD) Research Framework highlights the web of influences including individual, interpersonal, community, and societal factors constructing and perpetuating health inequities. The Social Determinants of Health (SDOH), central to these inequities, encompass the broader conditions impacting individuals’ lives — where they grow, live, work, play, and age — and societal political and economic forces. Studies have consistently documented the impact of factors interacting across multiple levels, pointing to the potential for Multilevel Interventions (MLIs) to improve health equity-oriented outcomes (Alvidrez et al., 2019; Agurs-Collins et al., 2019). Despite their promise, methodological challenges continue to limit the development of MLIs targeting multiple SDOH (Agurs-Collins et al., 2019).

This paper introduces the Multilevel Intervention Stepped Wedge Design (MLI-SWD), a hybrid randomized design for examining MLIs that involve both Individual Level (IL) and Cluster Level (CL) interventions. The MLI-SWD combines directly randomizing individual participants to IL treatments with a Stepped Wedge Design (SWD) for assigning CL treatments. This design enables estimation of the both component and overall treatment effects. Building on recent work in sample size estimation for SWDs using Generalized Estimating Equations (GEE) (Liang & Zeger, 1986; Zhang et al., 2023), we extend this approach to the multilevel case and provide an R package for performing these calculations. We explore potential model parameterizations, including how they affect the substantive interpretation of the parameters, and present a detailed example of a sample size calculation for a hypothetical trial. We discuss the example study’s most salient design decisions and their rationale. Before closing, we discuss adapting the design for scenarios with incomplete observation patterns and when individuals join clusters during the study.

## Background

Guided by social-ecological models such as the NIMHD Research Framework, MLIs are increasingly recognized as holding promise for addressing SDOH. Developing MLIs requires additional consideration for how determinants (and interventions) at one level can modify the effects of determinants (and interventions) at another (Weiner et al., 2012). Intervening at multiple levels can remove barriers that would otherwise prevent an intervention from being effective, but it can also create frictions that make other components less effective. Yet MLIs are often developed by combining components that have been validated separately, and the resulting MLI evaluated in a randomized experiment with the entire intervention package as the treatment precluding evaluation of the individual components (Collins et al., 2005; Collins, 2018). Collins et al. (2005) proposed the Multiphase Optimization Strategy (MOST) framework as an alternative way to develop behavioral interventions, including MLIs, using multiple phases of experimentation. The first phase consists of screening a set of potential components to identify a promising subset using a design that can efficiently test numerous treatments such as a factorial design. The promising interventions are then experimentally refined in the spirit of dose-finding trials, and finally the MLI is assessed in a Randomized Controlled Trial (RCT) (Collins et al., 2007; Collins, 2018). While MOST emphasizes screening numerous potential intervention components, it does not address randomizing interventions at different hierarchical levels. Approaches to address preexisting clustering exist, but only consider completely cluster randomized or individually randomized designs (Dziak et al., 2012).

### The North Carolina Works 4 Health Study

The North Carolina Works for Health (NCW4H) Shawn (2024) project aims to develop and evaluate an MLI for reducing the negative health consequences stemming from unemployment. In phase one, the study team worked with community partners and stakeholders (including people who are Socioeconomically Disadvantaged (SED) and unemployed) to adapt evidence-based interventions to reflect the experiences of unemployed SED groups while maintaining core intervention components. At the IL, the Diabetes Prevention Program was modified to develop the Chronic Disease Prevention Program (CDPP) — a lifestyle and behavior change intervention focusing on managing stress related to unemployment, building problem-solving skills, and using coping styles that promote healthy behaviors. The CDPP includes online content modules, face-to-face sessions with a lifestyle coach for individualized goal-setting and activity planning, and a system of stepped care for monitoring goals. At the CL, the *Supervising for Success* (*S4S*) program was adapted from an implicit bias habit-breaking intervention, the Prejudice Habit Breaking Intervention (Cox & Devine, 2019), to focus on enhancing supervisor support for SED hires. Supervisors take an interactive online training course on implicit bias and employment-related challenges associated with resource deprivation (e.g., potential lack of reliable transportation). The CL intervention also includes setting up weekly 5-minute check-ins between them and their new hires for 8 weeks and then bi-weekly thereafter.

The NCW4H was planned and launched as a $2\times 2$ factorial design with randomization to parallel groups at the IL and CL. The CL recruitment targets employers, and to incentivize enrollment the S4S program is offered to all of their supervisors. Recruitment began in September 2021, and employer recruitment proved exceedingly difficult. Discussions with local employers identified the time before receiving the intervention when in the control condition as the most significant barrier to participation. Initially, the CL component was scheduled to be given to all employers at the completion of the study, but employers reported viewing this as too distant while enrolling would require administrative effort up-front. While the NCW4H study inspired the current design, we defer discussing it until the "Extensions" section because it required additional adaptations that aren't necessary for the MLI-SWD with a complete design.

### Stepped Wedge Designs (SWDs)

A SWD is a type of crossover design characterized by unidirectional switching from control to intervention with random assignment of the switch timing. SWDs are fundamentally a pragmatic design because treatment effects are confounded with calendar time by design (Hussey & Hughes, 2007). Most commonly used in cluster-randomized trials (CRTs), SWDs have experienced an explosive growth in popularity over the past decade leading to the 2018 extension to the Consolidated Standards of Reporting Trials (CONSORT) guidelines specific to SWDs (Hemming et al., 2018). Their growth has also generated controversy because of their greater vulnerability to time-based confounding (Murray et al., 2020). SWDs potentially permit randomization where it was previously implausible, namely in settings where delivering the intervention to all treated clusters simultaneously is logistically challenging. Logistical constraints may stem from relying on trained study personnel to physically deliver the intervention, such as with in-person training, or when the implementation is time-consuming as can occur with interventions that change operating procedures at institutions such as municipalities, hospitals, or schools — constraints commonly encountered in developing MLIs (Hemming et al., 2015; Agurs-Collins et al., 2019).

**Fig. 1:**
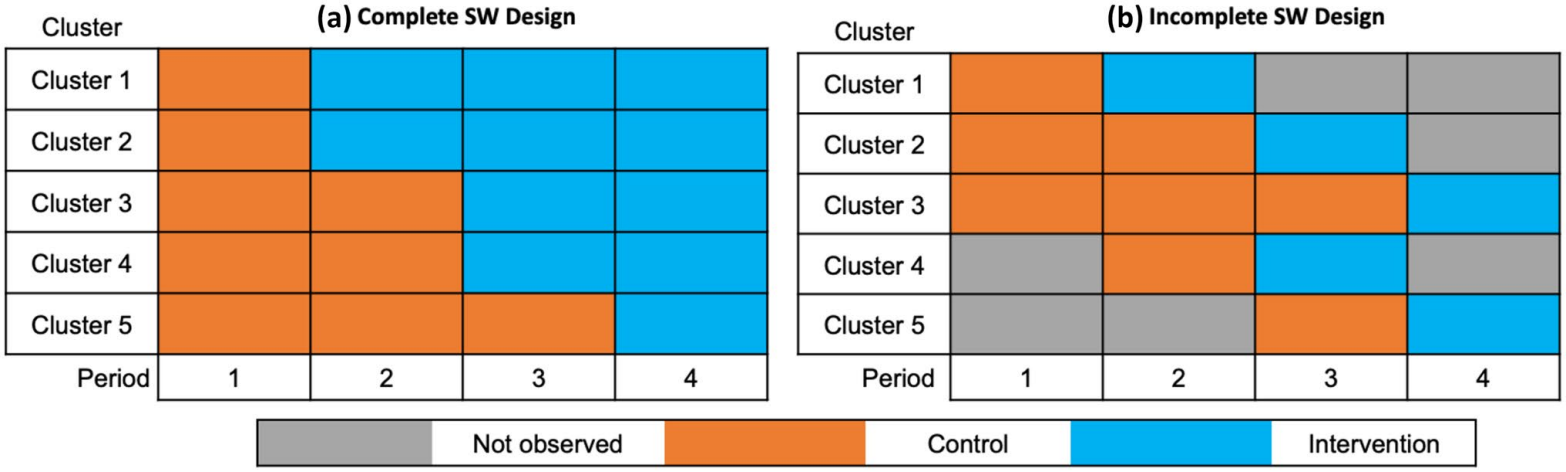
Example four-period complete and incomplete stepped wedge designs

SWDs can be complete (all clusters are observed at every time point) or incomplete (clusters may have unobserved periods), and may collect data from the individuals within the study clusters using a cohort (same individuals each time) or cross-section (new sample at each time). We introduce our design by considering closed cohort, complete designs before detailing extensions for incomplete and open-cohort designs which carry additional design considerations. Incomplete SWDs have periods where different clusters will be unobserved *by design* by constraints including 1) staggered enrollment (and consequently study completion) and 2) the need for an implementation period when transitioning from the control condition to the intervention. Figure 1a shows examples of a four-period complete SWD, and Fig. 1b presents an incomplete SWD with no implementation periods where study participation ends after one time period on the intervention. We see that clusters may enter the study at different times (and end participation at different times), and that clusters may be followed for different lengths of time depending on the design. Incomplete designs may recruit all clusters before the study begins with randomization defining their observation schedule. Staggered enrollment (incomplete) SWDs are typically treated as distinct from continuous enrollment SWDs because of distinct design considerations (Hooper & Copas, 2019), but this distinction can be blurry in practice. Our design and formulae accommodate complete and incomplete designs as well as cohort and cross-sectional samples.

#### Determining if a Stepped Wedge Design is Appropriate

SWDs have been an amazing development in experimental design due to enabling randomization in challenging contexts, but they are not a panacea. “The chosen design should be appropriate for the study’s context” is a truism, but SWDs require particularly careful consideration due to their inherent susceptibility to time-based confounded (Hemming et al., 2015; Zhang et al., 2023). Fortunately, detailed guidance is available to help researchers with these questions (Hemming et al., 2020a; Murray et al., 2020). Common rationales for choosing a SWD — logistical feasibility, improved power, recruitment benefits, and ethical or political constraints in withholding interventions — are the subject of ongoing debate (Beard et al., 2015; Hooper & Eldridge, 2021). There are undoubtedly situations where SWDs permit randomization where it would otherwise be impossible, but their purported benefits in other cases are situational. SWDs can sometimes create additional logistical challenges, power is contingent on study characteristics, recruitment differences have not been empirically tested, and parallel group designs can offer the intervention at the end of the study to satisfy concerns related to withholding the intervention (Murray et al., 2020). Power differences between parallel group designs and SWDs vary depending on the sampling design (cohort or cross-sectional) the structure of the crossover timings, the number and variability of clusters and cluster sizes, and the estimand of interest among other factors (Baio et al., 2015; Hemming & Taljaard, 2020). Cohorts may be preferable for studying MLIs for statistical efficiency. When a SWD has higher power than a parallel groups design, a crossover design where some units also switch from treatment to control, not just control to treatment, may further improve power without the same susceptibility to time-related confounding (Hemming et al., 2020a, b).

The decision to use a SWD should primarily be guided by practical rather than statistical considerations because their vulnerability to time-varying confounders is not shared by other common CRT designs. The risk posed by time-varying confounders can be mitigated, though not eliminated, through appropriate analytic choices. Our experience with the NCW4H study illustrates this point. The decision to change mid-study to a SWD was driven by the need to substantially improve employer recruitment. Recruitment improved, but caution should be taken extrapolating from this experience. COVID-19 created numerous challenges for employers in 2021 that may have influenced recruitment regardless of study design. There is also the possibility that reducing the wait time for the intervention for control group employers might have sufficed to address recruitment issues.

#### Analyzing Stepped Wedge Trials

Stepped Wedge Trials (SWTs) require statistical methods that account for the correlation between units in the same cluster and, with cohort designs, between repeated observations on the same units over time. Analyzing longitudinal, or panel, data requires careful attention to its temporal nature particularly when covariates can vary over time (Diggle, 2002; Hsiao, 2022). The two primary approaches to modeling SWTs are frequentist random effects models (mixed models), and frequentist marginal approaches estimated using GEE (Liang & Zeger, 1986). While uncommon, Bayesian Hierarchical Models (Gelman & Hill, 2006) are also appropriate for analyzing SWTs. We adapt an approach based on GEE for sample size calculations (Li et al., 2018; Zhang et al., 2023) to MLIs. GEE is attractive in our setting because the parameters in marginal models have a straightforward interpretation in terms of how the population average individual response changes — each parameter corresponds to the increase in the expected value on the scale of the link function associated with a one-unit change in the associated covariate assuming that all other covariates are held constant, and as such are closely aligned with policy-making goals. In contrast, the parameters in mixed effects models have an interpretation conditional on the (unobservable) random effects — e.g., “for a cluster with a random intercept value of *x*, each parameter corresponds to the increase in the expected value on the scale of the link function associated with a one-unit change in the associated covariate.” In nonlinear mixed models these conditional parameters are not generally equal to the marginal parameters.

Clear specification of the target estimand(s) is particularly important in CRTs with repeated measurements because there are choices between both marginal or conditional estimands, between participant- or cluster-average estimands, and summaries of time-varying treatment effects (Kahan et al., 2023). Kahan et al. (2023) demonstrate that the resulting estimates for different estimands can vary greatly and provide guidance for the choosing and reporting estimands. We estimate *marginal participant-average treatment effects*, i.e., marginal rather than conditional and participant-average rather than cluster-average. Different summaries of time-varying treatment effects can be targeted by the choice of mean model parameterization, which change the meaning of the estimated coefficients, and contrasts for hypothesis tests. However, there are potential time-varying-related estimands not covered by our approach, notably functional estimands including the area under the curve; see Kenny et al. (2022) for a discussion of other potential time-related estimands. To fully specify the estimand one also needs to define the target comparison such as a risk difference or odds ratio, and the choice of target informs what link function should be used. For example, when the response is binary, choosing the identity link provides parameters that can be interpreted as risk differences, while choosing the logistic link results in parameters interpreted as odds ratios.

Finally, we wish to emphasize that the model we present for sample size calculations is simplified compared to what we would suggest for analyzing a completed study. The sample size formula we provide can be used with categorical, linear, and general polynomial time trends, but in the actual analysis combining GEE with smoothing splines or kernel regression for the time trend may be desirable. Splines and kernels are more robust than linear or polynomial models to misspecification, and they typically require fewer parameters (and cannot possibly use more than) than treating time as categorical (Welsh et al., 2002; Wang et al., 2005). While splines and kernels are difficult to directly interpret, the global time trend is typically a nuisance rather than of independent interest, and analysts can retain the benefits of having a parametric model for the intervention effects, such as interpretability, by only using the flexible model for the time trend. Similarly, when cluster size might be informative, an independence working correlation structure can be used as the working correlation model and the dependence corrected for with robust “sandwich” standard errors (Hemming & Taljaard, 2020; Sullivan Pepe & Anderson, 1994). There have also been recent developments in inferential methods (distinct from GEE) that are robust to confounding by time (Hughes et al., 2020; Wang et al., 2023) which can serve as the primary analysis or a sanity check on model-based estimates. If serving as the primary analysis, a simulation study should be conducted to confirm the desired power is reached.

## The Multilevel Intervention Stepped Wedge Design (MLI-SWD)

**Fig. 2:**
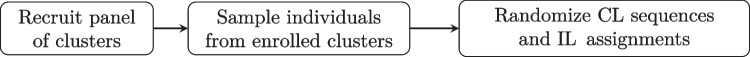
Randomization flow with existing nesting

We propose combining unit-level randomization at the individual level with a stepped wedge design at the cluster level. The IL assignment and the CL assignment to a transition time can be conducted using existing methods such as permuted block randomization. The SWD component permits complete and incomplete observation patterns and cross-sectional or cohort samples. Figure 2 shows the high-level process flow for the design’s randomization when individuals are nested within clusters at the start of the study. A cohort or cross-sectional sample can be formed by randomly sampling individuals within the enrolled clusters. At the start of the study, all clusters are randomized to a treatment sequence determining when they begin the CL intervention, and individuals are concurrently randomized to an IL condition.

Randomizing the IL intervention individually can bring substantial efficiency benefits over randomizing it at the CL (so that everyone in the same cluster has the same IL intervention). This benefit manifests in reducing the variance of not only the IL treatment effect estimate, but estimates of the interaction effect and the overall intervention effect as well. However, if the combined intervention effect is solely of interest, a design that only randomizes clusters between no intervention and receiving both the IL and CL may be more powerful than the MLI-SWD depending on the correlation structure, and it will be simpler.

### When Individual-Level Randomization Should Not Be Used

Researchers should weigh the severity of the risk of spillover effects or contamination at the IL when choosing whether to randomize at both the IL and CL. Randomizing at the CL only avoids the issue of spillover at the IL because all individuals in the same cluster would receive the same IL intervention. CL contamination is a threat to both randomization schemes. The plausibility of the no spillover assumption depends on the specifics of the interventions, individuals, and clusters. For example, an IL training intervention may be at low risk of spillover when clusters are cities but high risk if the clusters are families. If this assumption is implausible, then the IL intervention should be cluster randomized. However, some level of spillover is likely tolerable — with low-to-moderate spillover and large samples, standard estimators are consistent for the expected average treatment effect (Sävje et al., 2021; Hemming et al., 2021).

## Data Generating and Analysis Models

Let $i= 1, \ldots , I$ index clusters, $k= 1, \ldots , K$ index individuals, and $j= 1,\ldots , J$ index the discrete time periods at which outcomes are measured. The calendar time period corresponding to the *j*th observation on individual $k$ in cluster $i$ is denoted by $T_{ijk}$. Let $A_{ijk}^{\text {IL}}$ denote the number of time periods that individual $k$ has been on the IL intervention, and let $A_{ijk}^{\text {CL}}$ denote the number of time periods cluster $i$ containing individual $k$ has been on the CL intervention including period *j*. Individual $k$’s outcome in period *j* is denoted by $Y_{ijk}$.

We first define the mean model in its general form to highlight the level of choice available to the researcher before showing concrete examples of common definitions. Denote the sample space by $\mathcal {X}$, and let $\varvec{f}: \mathcal {X}\mapsto \mathbb {R}^{d}$ be a fixed and known function which creates the design matrix which specifies the covariates in the model. The model for the expected response $\mu$ for individual $k$ in cluster $i$ at observation time $j$ is given in its general form in Eq. 1 on the scale of the link function ${g}$.

1 $$\begin{aligned} \displaystyle {g}(\mu _{ijk}) = \varvec{f}\left( T_{ijk}, \, A_{ijk}^{\text {IL}}, \, A_{ijk}^{\text {CL}}, (A_{ijk}^{\text {IL}}, A_{ijk}^{\text {CL}}) \right) \varvec{\theta }\end{aligned}$$

The researcher can decompose the function $\varvec{f}$ into a set of component functions that define particular aspects of the design matrix. For example, the decomposition $\varvec{f}= \left( f_{T}(T_{ijk}),\, f_{\text {IL}}(A_{ijk}^{\text {IL}}),\, f_{\text {CL}}(A_{ijk}^{\text {CL}}), \, f_{\text {Int}}(A_{ijk}^{\text {IL}}, \, A_{ijk}^{\text {CL}}), \ldots \right)$ includes functions defining the elements associated with the calendar time, the IL, CL, and interaction ($\text {Int}$) effects respectively; the ellipses represent the potential for the design function to include other covariates. The functions $f_{T}, f_{\text {IL}}, \,f_{\text {CL}},\, f_{\text {Int}}$ can specify different models with distinct interpretations of the associated treatment effect parameters. Let ${{\,\mathrm{{\textbf{1}}}\,}}$ be the indicator function which is equal to one when its condition is satisfied and zero otherwise. The expected intervention effects can be specified to not vary with the time on treatment, sometimes called the “average intervention effects model” (AIM), or a linear relationship between the time on treatment and the response could be used (the “incremental intervention effects” (IIM) model) (Ouyang et al., 2022). Table 1 defines the IL, CL, and interaction term functions $f_{\text {IL}}, f_{\text {CL}}, f_{\text {Int}}$ for both of these models.

**Table 1:** Common mean model intervention effect definitions

Effects model	$f_{\text {IL}}(A_{ijk}^{\text {IL}})$	$f_{\text {CL}}(A_{ijk}^{\text {CL}})$	$f_{\text {Int}}(A_{ijk}^{\text {IL}}, A_{ijk}^{\text {CL}})$
Average	${\textbf {1}}(A_{ijk}^{\text {IL}}\ge 1)$	${\textbf {1}}(A_{ijk}^{\text {CL}}\ge 1)$	${{\,\mathrm{{\textbf{1}}}\,}}\left\{ A_{ijk}^{\text {IL}}\ge 1 \right\} {{\,\mathrm{{\textbf{1}}}\,}}\left\{ A_{ijk}^{\text {CL}}\ge 1 \right\}$
Incremental	$A_{ijk}^{\text {IL}}/ c^{\text {IL}}$	$A_{ijk}^{\text {CL}}/ c^{\text {CL}}$	$\frac{1}{c^{\text {Int}}} \sum _{j\prime = 1}^{k} {{\,\mathrm{{\textbf{1}}}\,}}\left\{ A^{\text {IL}}_{ij\prime k} \ge 1 \right\} {{\,\mathrm{{\textbf{1}}}\,}}\left\{ A^{\text {CL}}_{ij\prime k} \ge 1 \right\}$

In the incremental model $c^{\text {IL}}, c^{\text {CL}}, c^{\text {Int}}$ are constants chosen to scale the intervention effect to give the interpretation of the treatment effect parameter meaning at a scientifically relevant time point. For example, when $c^{\text {CL}} = 3$, $\delta ^{\text {CL}}$ is the CL intervention effect on the scale of the link function after 3 time periods on the CL intervention. Here $f_{\text {Int}}$ is chosen so that it is linear in the number of time periods on both IL and CL interventions; using the product $A_{ijk}^{\text {IL}}A_{ijk}^{\text {CL}}$ would be quadratic rather than linear in calendar time. Other choices of $\varvec{f}$ are possible such as quadratic models but not discussed here. Similarly, the choice of $f_{T}$ can specify different effects of calendar time. Common choices include a linear time trend and treating the time periods as categorical so that every period has an associated indicator variable (depending on the parameterization the first period may be included in an intercept term). Equation 2 contains an example parameterization of the AIM with categorical time periods.

2 $$\begin{aligned} \displaystyle {g}(\mu _{ijk}) &= T_{ij} \beta _{t} + {{\,\mathrm{{\textbf{1}}}\,}}\left\{ A_{ijk}^{\text {IL}}\ge 1 \right\} \delta ^{\text {IL}}+ {{\,\mathrm{{\textbf{1}}}\,}}\left\{ A_{ijk}^{\text {CL}}\ge 1 \right\} \delta ^{\text {CL}} \\&\quad+ {{\,\mathrm{{\textbf{1}}}\,}}\left\{ A_{ijk}^{\text {IL}}\ge 1 \right\} {{\,\mathrm{{\textbf{1}}}\,}}\left\{ A_{ijk}^{\text {CL}}\ge 1 \right\} \delta ^{\text {Int}}\end{aligned}$$

Equation 3 is an example of the IIM with a linear time trend ($A_{ijk}^{\text {Int}}$ is as defined in Table 1).

3 $$\begin{aligned} \displaystyle {g}(\mu _{ijk}) =\beta _0 + T_{ij}\beta _1 + A_{ijk}^{\text {IL}}\delta ^{\text {IL}}+ A_{ijk}^{\text {CL}}\delta ^{\text {CL}}+ A_{ijk}^{\text {Int}} \delta ^{\text {Int}}\end{aligned}$$

There is a bias-variance trade-off to consider when specifying time trends. Categorical period effects allow maximum flexibility at the cost of requiring as many parameters as time periods. A single-parameter linear specification is almost certainly misspecified, but it may be a reasonable approximation if large changes in the trend are unlikely during the study window. A misspecified linear model can have a lower mean squared error than the correctly specified nonlinear model if the region of function being estimated is approximately linear because the increased variance from estimating more parameters can outweigh the reduction in bias (Gelman, 2000). It is worth reiterating here that a more complex model can be used for analysis after using a simplified model for sample size calculation.

### Variance Specification

In addition to specifying an outcome model, GEE also requires specifying the variance and the working correlation structure between observations within a cluster over time. The variance is modeled by $\textrm{V}(Y_{ijk}) = \phi v_{ijk}(\mu _{ijk})$ where $v_{ijk}(\mu _{ijk})$ is the variance as a function of the mean and $\phi$ is a dispersion parameter. The mean model dictates the variance model; for a normal model the dispersion parameter is the variance $\phi = \sigma ^2$ and the variance function $v_{ijk}(\mu _{ijk}) = 1$ because the variance of a Gaussian random variable is not a function of the mean. For the standard logistic regression model, it is assumed that there is no overdispersion so $\phi = 1$, while the variance is a function of the mean namely $v_{ijk} = \mu _{ijk}(1-\mu _{ijk})$. The working correlation matrix $R_i$ is parameterized by a vector $\varvec{\alpha }$ that defines the relationship between observations in a cluster; the meaning of these parameters depends on the correlation structure that is specified. The results of Li et al. (2018), Zhang et al. (2023) and our modifications apply to both block exchangeable and autoregressive correlation structures. The block exchangeable structure includes parameters for the within-period correlation (different people, same time), inter-period correlation (different people, different times), and within-individual correlation (same person, different times).

### Small Numbers of Clusters

SWDs are often employed in studies with a small number of clusters. GEE is asymptotically unbiased, but confidence intervals based on asymptotic approximations can have poor coverage with small samples. The *t*-distribution, with heavier tails than the normal, can help reflect this greater uncertainty in sample size calculations. The degrees of freedom can be set by the number of clusters $I$ minus the dimension of the mean model parameter vector $\varvec{\theta }$ (five in our example parameterization); other estimators may improve performance but are more complex (Fay & Graubard, 2001). Correction factors (Kauermann & Carroll, 2001) and model building for the correlation parameters (Preisser et al., 2008) may aid analysis; interested readers can consult Thompson et al. (2021) for simulation studies of finite-sample adjustments.

## Power and Sample Size Calculations

Our power and sample size calculations are based on the Wald test following Rochon (1998) and Zhang et al. (2023). Let $L$ be a $q \times d$ matrix where $d= \dim (\varvec{\theta })$ and *q* is the number of constraints imposed by the hypothesis we wish to test. For example, a test of the null hypothesis that the IL effect is zero, $\delta ^{\text {IL}}= 0$, imposes one constraint namely $\delta ^{\text {IL}}= 0$ and so $q = 1$. We will specify all of our hypotheses in terms of $H_0: L\varvec{\theta }= \varvec{\ell }$ vs $H_1: L\varvec{\theta }\ne \varvec{\ell }$ where $\varvec{\ell }$ is a *q*-dimensional vector of constants corresponding to the parameter values under the null. The null values $\varvec{\ell }$ are typically zero or a zero vector, but any constants could be specified provided there is a scientific justification for their choice. Asymptotically $\sqrt{I}(\widehat{\varvec{\theta }} - \varvec{\theta }) {{\,\mathrm{\underset{d}{\rightarrow }}\,}}N_{d}(0, \Sigma )$ (Balan & Schiopu-Kratina, 2005; Li et al., 2018). The Wald test statistic $W_I$ is then given by $(L\widehat{\varvec{\theta }} - \varvec{\ell })^{\textrm{T}} [L\widehat{\Sigma }_1^{-1}/IL]^{-1} (L\widehat{\varvec{\theta }} - \varvec{\ell })$ where $\widehat{\Sigma }_1$ is the model-based variance estimator and $I$ is the number of clusters (not an identity matrix). The Wald test statistic under the null asymptotically has a $\chi _q^2$ distribution and under the alternative it has a non-central $\chi _{q, \, \lambda }^2$ with noncentrality parameter $\lambda$. The characteristics of the study that are needed to perform the sample size calculation are detailed in Table 2, and the algorithm for computing the power for complete designs with closed cohorts or cross-sectional samples is detailed in Algorithm 1. The calculation for incomplete designs is the same except the design and working correlation matrices are replaced with incomplete versions that account for the missingness patterns; these modifications are discussed in the "Extensions" section.

**Table 2:** Study characteristics — complete design

**Parameter**	**Name**	**Description**
$T = J$	Time periods	Total number of study time periods
*S*, $m_s$	Sequences	Number of unique sequences *S*, and the number of clusters in each sequence $m_s$. The total number of clusters is $I = \sum _{s} m_s$
$N_{ia^{\text {IL}}}$	Individuals	Number of individuals with IL treatment $a^{\text {IL}}$ in cluster $i$. The total number of observations in cluster *i* is $N_{i0} + N_{i1}$
$${\textbf {X}}_i = \left[ \begin{array}{c} X_{i(a^{\text {IL}}= 0)} \\ X_{i(a^{\text {IL}}= 1)} \end{array} \right]$$	Design matrices	Cluster-level design matrices for each cluster where there is one row per observation per individual
$\varvec{\theta }$	Mean model	Coefficient values of the parameter vector for the mean model, i.e., the time and treatment effects
${g}$	Link function	Link function for the mean model. It is determined by the primary outcome and the estimand of interest, e.g., logit link for the odds ratio of a binary response
$v_{ijk}(\mu _{ijk})$, $\phi$	Variance	Variance components: the variance as a function of the mean (expected response), and the dispersion parameter $\phi$
$\varvec{\alpha }$	Working Correlation	Parameter vector for the working correlation matrix
$L$, $\varvec{\ell }$	Contrasts	Null hypotheses defined in terms of contrasts between parameters. Determined by scientific questions and estimand

Potential sample sizes can be determined for a given power by searching over potential values of the number of clusters and number of individuals per cluster as inputs to the power calculation. There will be multiple combinations of the number of individuals and the number of clusters that can achieve a desired power. We have developed an R package for performing both power and sample size calculations https://github.com/jsperger/swtgeepower. The package can be used to perform all of the calculations detailed in this paper for continuous, binary, and count outcomes with identity, log, and logistic link functions. The package is still under active development to add features and improve its accessibility to nonstatisticians.

**Algorithm 1** GEE power calculation for multilevel stepped wedge design
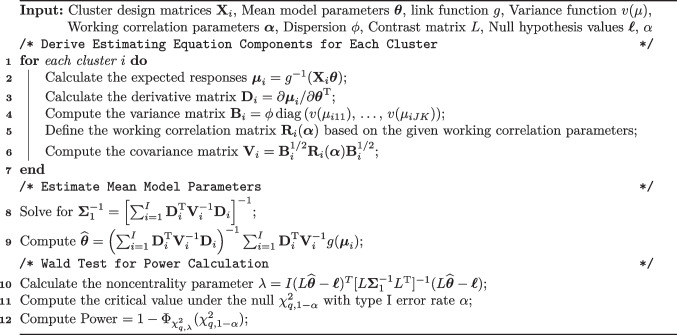


### Example Parameterization and Power Calculation

We will now walk through an example to demonstrate specifying a data generating model, choosing an estimand, and performing a power calculation. Suppose a state is interested in a potential public health intervention to improve diabetes-related outcomes in small towns and rural areas with a multi-level intervention that includes education and coaching for individuals while providing towns with funding to give ride vouchers to community members with diabetes. The individuals are already nested within clusters and all clusters and individuals will be recruited before the study begins. The planned design will be a complete, closed-cohort design. The coaching one individual receives is unlikely to have a great effect on other people in the same town or city (cluster), and providing ride vouchers could address a significant barrier to accessing care which is potentially synergistic with individual coaching and education. The primary outcome is the average HbA1c level in the past week measured by a study provided continuous glucose monitor. The study’s primary aim is to evaluate the effectiveness of the overall intervention package, and its secondary aim is to evaluate the effectiveness of each component intervention.

**Fig. 3:**
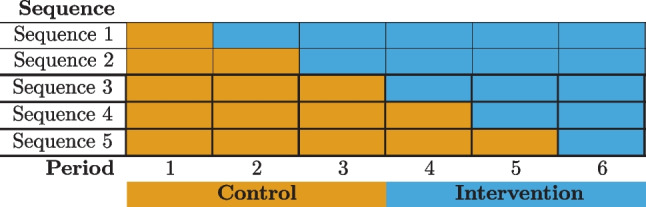
Six-period complete stepped wedge design

The investigators decide based on logistical considerations that they can take measurements and roll the cluster-level intervention out to a new wave every two months, and they can run the study for one year. The number of time periods is $T = 6$ and the number of unique sequences $S = 5$ because everyone clusters only begin to cross over in period two. The schematic for such a design is given in Fig. 3. A linear time trend may be a reasonable approximation of any potential temporal trends here because the time periods are short and the primary outcome at the population level is generally stable. Similarly, utilization and allowing time for changing habits to take effect suggest a time-varying treatment effect, and a linear approximation is thought to be reasonable given the time frame of the study. If the study ran for longer periods of time this would be unlikely. Thus the study planners decide that the incremental intervention effects model with linear effects of calendar time and time on treatment for both IL and CL are appropriate. They are interested in the individual-level population average difference in HbA1c after six months on the combined intervention so they scale the treatment time covariates in the design matrices by 1/3, the number of study periods corresponding to six months, for the parameters to have the desired interpretation. The primary outcome $Y_{ijk}$ can be treated as approximately continuous, and with the target estimand implies choosing the identity link function ${g}(x) = x$.The mean model parameters based on these decisions are $(\beta _0, \beta _1, \delta ^{\text {IL}}, \delta ^{\text {CL}}, \delta ^{\text {Int}}) = \varvec{\theta }$.

The investigators want to ensure power for both their primary and secondary aims after adjusting for multiple comparisons with the Bonferroni correction. The primary aim corresponds to testing the combined intervention effect $H_0: \delta ^{\text {IL}}+ \delta ^{\text {CL}}+ \delta ^{\text {Int}}= 0$ vs. $H_1:\delta ^{\text {IL}}+ \delta ^{\text {CL}}+ \delta ^{\text {Int}}\ne 0$ which is given by contrast vector $$L= \begin{bmatrix} 0&0&1&1&1 \end{bmatrix}$$. The secondary aim corresponds to two hypothesis tests with the null hypotheses $H_0: \delta ^{\text {IL}}= 0$ and $H_0: \delta ^{\text {CL}}= 0$.: They desire 80% power for all tests with $\alpha =.05$ for the primary aim and $\alpha =.1$ for the secondary aim’s tests. Reviewing the literature, their best guess of the standardized effect size of each component is $\delta ^{\text {IL}}= 0.1$ for the IL intervention and $\delta ^{\text {CL}}= 0.15$ for the CL intervention. There were no studies of their combined effect to guide specifying the interaction term, so they decided to set the interaction term based on the minimum effect size for the combined intervention that they considered to be meaningful. Given the high health impacts of diabetes, they decide that a standardized effect size of at least 0.15 would be meaningful; combining this with the component effect estimates they calculate $\delta ^{\text {Int}}= -0.1$.

After specifying the mean model, they next define the correlation structure. Because the study follows a closed cohort, the investigators decide between using a block exchangeable correlation structure or a corresponding autoregressive structure. The investigators believe that while the correlation between repeated HbA1c measurements likely decays over time and has an autoregressive correlation structure on a minute-by-minute basis, when measurements are taken every two months over the course of one year they are likely to be equally correlated. Thus they choose to use a block exchangeable correlation structure, and find published estimates of $\varvec{\alpha } = (.05,.025,.5)$ for the within-period, between-individual-and-period, and individual-between-period correlations respectively.

When planning a study there may be practical constraints on the number of clusters or the number of individuals per cluster. If there are not these constraints, investigators can calculate a frontier of possible combinations of cluster and individual sample sizes to achieve the desired power and choose based on some other criteria. Here the investigators consider $I = 65$ towns overall, $m_s = 13$ per sequence, as a reasonable limit on the number of clusters, while they can enroll as many individuals per cluster as needed within reason. To achieve at least 80% power after adjusting for multiple comparisons for all three hypothesis tests that comprise the primary and secondary aims, the study would need to enroll $N_i = 15$ individuals per cluster for a total of 975 people. With this sample size, the anticipated power for the test of the intervention overall is approximately 81%, the test of $\delta ^{\text {IL}}$ 95%, and the test of $\delta ^{\text {CL}}$ 80%; there would only be 40% power to test $\delta ^{\text {Int}}$ at the same level as the $\delta ^{\text {IL}}$ and $\delta ^{\text {CL}}$.

This example was simplified for exposition. Loss to follow up was ignored, and, at a minimum, the investigators should perform sensitivity analyses with unequal cluster sizes and different correlation values. The assumption about the correlation between observations on the same individual is often especially influential with cohorts.

## Extensions

### Incomplete Designs

In transitioning to incomplete designs, we now need to distinguish between observation time *j* and calendar time *t*. Observation time *j* refers to the periods a cluster *i* is observed, starting at 1 for each cluster regardless of when they enter the study. The number of observations on a cluster, $J_i$, may vary if clusters are observed for different lengths of time. In contrast, the calendar time *t* indexes the entire study periods, ranging from 1 to *T*.

Specifying an incomplete design begins with creating the complete design and then defining the patterns of planned missingness for each sequence. Note that the missingness in an incomplete design is planned, and the procedure here should not be used to analyze a study with missing cluster observations. An incidence matrix $K_i$ is created for each cluster *i* to represent the observation pattern. $K_i$ maps observations to calendar time periods, with the columns of $K_i$ corresponding to calendar time periods, and each row of $K_i$ corresponding to a single observation within the cluster. Each row of $K_i$ contains a single ‘1’ to designate that the observation was observed in that calendar time period with zeros in all other positions. The incidence matrix is used to transform the complete design matrix ${\textbf {X}}_i$ into its incomplete counterpart ${\textbf {X}}_{i}^{\text {inc}} = K_i {\textbf {X}}_{i}$, as well as the working correlation matrix $R_i(\varvec{\alpha })^{\text {inc}} = K_i R_i$. In a complete design with a closed cohort, $K_i$ would have $N_iJ$ rows reflecting that all $N_i$ individuals are observed for all $J = T$ time periods.

The process to construct $K_i$ differs based on the type of cohort. In cross-sectional and closed-cohort designs, constructing a representative incidence matrix $K_s$ for a single individual in the cluster is sufficient. The cluster incidence matrix $K_i$ is obtained by $K_i = K_s \otimes \varvec{1}_{N_i}$ where $N_i$ is the number of individuals in the cluster and $\varvec{1}$ a column vector of ones. In an open cohort, the sub-component for each individual must be defined and concatenated to form the cluster-level incidence matrix in order to allow individuals to belong to a cluster for different lengths of time. Algorithm 1 can be used to calculate power for an incomplete design by substituting the incomplete design matrices ${\textbf {X}}_{i}^{\text {inc}} = K_i{\textbf {X}}_{i}^{\text {comp}}$ and correlation matrices ${\textbf {R}}_{i}^{\text {inc}} = K_i{\textbf {R}}_{i}^{\text {comp}}$ for their complete counterparts.

### Allowing Individuals to Begin Outside of Clusters

**Fig. 4:**
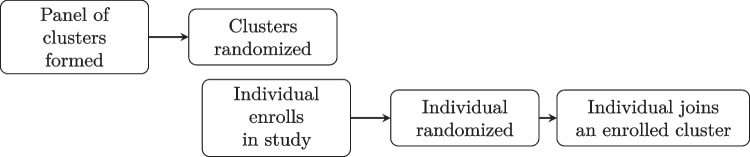
Randomization flows when individuals begin outside of clusters

The design could be applied in settings individuals do not start nested within clusters but join them during the course of the study though additional resumptions are required; the high-level process for this case is shown in Fig. 4. The NCW4H inspired this use case because the IL component is delivered while an individual is unemployed, whereas the CL would only be given after a study individual was hired. The panel of clusters can be formed and randomized as before in Fig. 2, but individuals must be recruited separately rather than sampled from the clusters. Challenges in this setting that are not present when individuals begin in clusters include that individuals may join clusters based on the CL assignment, individuals will belong to clusters for varying amounts of time, and defining the time on a CL intervention is murkier because a cluster’s time on the CL treatment will not necessarily match the time that an individual has belonged to a treated cluster as it did in the complete design. These issues exist in the background of CRTS when it’s possible for individuals to join a cluster mid-study, but they are central when all individuals need to join a cluster by design. With respect to modeling, $f_{\text {CL}}(A_{ijk}^{\text {CL}})$ could define covariates for both the number of time periods that the cluster has been on the CL intervention and the number of time periods that the individual has belonged to a cluster on the CL. If the data is rich enough these will both be estimable, but they may be difficult to disentangle in small samples. For observations before an individual belongs to a study cluster, pseudo-clusters can be defined with their own working correlation structure and parameters. Pseudo-clusters can be meaningful, e.g., counties in NCW4H, or an analytical fiction, and the correlation structure should reflect this choice.

Estimating the causal effect of a time-varying exposure or intervention requires that the potential outcomes for all future time periods $j + 1, \ldots , J$ be conditionally independent of the current observed exposure at time *j* given the observed past history including covariates, treatment assignments, and responses up through and including time *j*. Proposition 7 in Robins et al. (1999) establishes a sufficient condition for GEE estimates to be causally valid in this setting, namely that the conditional assignment probability at both the IL and CL given the observed history and any time-varying covariates must be equal to the conditional assignment probability given the observed history excluding time-varying covariates. So long as this holds having individuals belong to clusters for different lengths of time does not bias treatment effect estimates. This condition holds for both interventions by virtue of randomization regardless of any potential unmeasured confounders when individuals begin in clusters and clusters are recruited before the study is launched, but it is an assumption when individuals begin outside of clusters. To see this, note that when all clusters and individuals are randomized to a treatment sequence concurrently at the start of the study the treatments at all time points are a deterministic function of the assigned sequence. Consequently the treatment values at all time points are conditionally independent from all other covariates given the assigned sequence. For this condition to hold in open cohort designs or when individuals do not initially start in a cluster the process by which an individual joins a cluster must be conditionally independent of its CL treatment assignment given the rest of the observed history. If this condition does not hold GEE is not appropriate, but alternative analysis methods may still be causally valid. For example, g-computation (Robins, 1986) could be used to model the conditional probability of joining a cluster, though requiring a model for the treatment assignment reflects the loss of a key benefit of randomization.

## Summary and Implications for Prevention Science

Research on designing MLIs is still in its infancy, and additional research on sample size calculation for time-varying treatment effects is an important future direction for both MLIs and SWDs generally (Kenny et al., 2022). Our approach can currently consider linear transformations of time-varying treatments parameterized as polynomial functions of the time on treatment, but not functional summaries like the AUC. Permitting an arbitrary number of levels and interventions at each level would expand the applicability of the MLI-SWD, and it may be possible to improve the efficiency of CL parameter estimation by incorporating cluster-total estimators (Su & Ding, 2021). We also did not address the issue of noncompliance which can be a common issue in CRTs, and are investigating if and how the causal assumptions related to individuals joining clusters could be loosened and tested.

Researchers working in prevention science understand the imperative to intervene on SDOH if we are to make any real progress in mitigating health inequities. In addition to taking a public health perspective and including cross-sector partnerships, developing and testing interventions across multiple levels is fundamental to these efforts (Hacker et al., 2021). While there have been conceptual advances in designing MLI that produce synergistic effects (Weiner et al., 2012), approaches to address the methodological challenges inherent in MLI designs remain (Agurs-Collins et al., 2019). As noted in the NCW4H study discussion, intervening on SDOH related to employment is inherently complex and dependent on the engagement of multiple actors across different sectors, settings, and time. MLI studies must adapt to these complexities, and we hope that introducing the MLI-SWD and providing software for sample size determination will enable more researchers to plan studies investigating MLIS that can disentangle the contribution of both the IL and CL components and investigate whether they are synergistic. The MLI-SWD offers more precise estimation of the IL intervention effect compared than an equivalent design where the IL intervention is also randomized at the cluster level by increasing the effective sample size for the IL intervention. If the effects of the IL and CL components are not of interest on their own, a simpler design may be more appropriate. The MLI-SWD has the potential to allow studies where individuals can be randomized before they are nested within a cluster though additional assumptions not typically needed in a randomized trial is necessary. When deciding whether to use the MLI-SWD researchers should pay particular attention to potential spillover effects at the IL because the MLI-SWD is vulnerable to bias in a way that is not a concern when all the individuals in a cluster receive the same IL intervention, and, like all SWDs, they should consider the plausibility of confounding by time (a risk that can be mitigated but not eliminated).
